# New York Tobacco Control Program Cessation Assistance: Costs, Benefits, and Effectiveness

**DOI:** 10.3390/ijerph10031037

**Published:** 2013-03-13

**Authors:** Sean A. Simpson, James M. Nonnemaker

**Affiliations:** RTI International, 3040 Cornwallis Road, P.O. Box 12194, Research Triangle Park, NC 27709, USA; E-Mail: jnonnemaker@rti.org

**Keywords:** cessation, tobacco policy, smoking

## Abstract

Tobacco use and cigarette smoking have long been causally linked to a wide variety of poor health outcomes, resulting in a number of public health policy initiatives to reduce prevalence and consumption. Benefits of these initiatives, however, have not been well-established quantitatively. Using 2005–2008 New York Adult Tobacco Survey data, we developed a simulation model to estimate the effectiveness and net benefits of the New York Tobacco Control Program’s (NY TCP’s) adult smoking cessation assistance initiatives, specifically media campaigns, telephone quitline counseling, and nicotine replacement therapy. In 2008, we estimate that NY TCP generated an estimated 49,195 additional, non-relapsing adult quits (95% CI: 19,878; 87,561) for a net benefit of over $800 million (95% CI: $211 million; $1,575 million). Although the simulation results varied considerably, reflecting uncertainty in the estimates and data, and data sufficient to establish definite causality are lacking, the cessation initiatives examined appear to yield substantial societal benefits. These benefits are of sufficient magnitude to fully offset expenditures not only on these initiatives, but on NY TCP as a whole.

## 1. Introduction

Tobacco use in general, and cigarette smoking in particular, has long been causally linked to a wide variety of health problems, such as cardiac and pulmonary conditions and various kinds of cancer, with concomitant losses of life years and productivity, dating back to the U.S. Surgeon General’s report on smoking and health in 1964 [1]. Since then, a range of federal and state policy initiatives and programs, including educational efforts, excise taxes, and cessation assistance, have resulted in substantial reductions in the prevalence of smoking. However, this decline has been slowing and perhaps leveling off; the Centers for Disease Control and Prevention (CDC) reported in 2011 [2] that 23.9% of adults smoked in 2005, declining only slightly to 21.5% in 2010.

Comprehensive state tobacco control programs began to appear in the late 1980s and early 1990s, and they have since expanded to all 50 states. CDC issued Best Practices for Comprehensive Tobacco Control Programs initially in 1999 [3], based on the experiences of the Massachusetts and California programs as well as the existing scientific literature; over the following years, funding for these programs increased dramatically, along with restructuring to match the recommendations of Best Practices. An updated Best Practices was released in 2007 [4], which reflected more recent findings in the literature and incorporated more information from state experiences; in particular, CDC highlights findings that link increases in tobacco control program expenditures to decreases in cigarette sales. 

The New York Tobacco Control Program (NY TCP) was established as part of the Health Care Reform Act of 2000. Its mission is to reduce tobacco-related morbidity and mortality and the social and economic burden caused by tobacco use. NY TCP funds, among other initiatives, Cessation Centers and the New York State Smokers’ Quitline; invests in media to encourage tobacco users to quit; and undertakes efforts to reduce patient costs for treatment. The New York State Smokers’ Quitline, established in 2000, provides telephone counseling, free 2-week nicotine replacement therapy (NRT) starter kits, an interactive Quitsite Web site, and a number of other services both directly to smokers and to health care providers.

No studies have directly examined the link between expenditures and cessation, but studies have examined the cost-effectiveness of tobacco control program interventions as a whole [5,6,7] and for specific components, as well as how program components interact with each other. Studies have examined three components: media campaigns, telephone quitlines, and state-funded NRT, typically provided for a fixed period to quitline callers.

Among studies relating to media, Farrelly *et al.* [8,9] examine the design and channels of advertising and media campaigns, and Mosbaek *et al.* [10] examine the general effectiveness of media campaigns in generating calls to quitlines. An *et al.* [11], Fellows *et al.* [12], Hollis *et al.* [13], Tinkelman *et al.* [14], Stead *et al.* [15], and Cummings *et al.* [16] examine NRT and quitline use, as well as the cost-effectiveness of both components, either jointly or separately. In general, they find that the introduction of free NRT through quitlines substantially and significantly increases call volume and that smokers who use NRT are significantly more likely to successfully quit. When the question is considered, these studies consistently find that the additional quits induced by providing free NRT result in a marginal cost-per-quit that is roughly the same as, and in some cases lower than, the marginal cost-per-quit prior to the introduction of free NRT.

In the current environment of public austerity and budget cutbacks, the benefit of state-funded cessation intervention is an eminently relevant topic. The current analysis examined three components of NY TCP: media (e.g., television advertisements), telephone quitline counseling, and NRT. The intent was to develop a model to estimate the effectiveness of these programs and, by extension, their benefits, to determine whether they outweigh the costs.

## 2. Methods

### 2.1. Data

The primary data source is the New York Adult Tobacco Survey (NY-ATS), a random-digit-dial telephone survey designed to be representative of adults aged 18 or older in New York State. The NY-ATS is fielded quarterly; data collection began in June 2003 and is ongoing as of this writing. The questionnaire includes measures of cigarette and other tobacco product use, smoking cessation, exposure to secondhand smoke, and related attitudes, beliefs, and intentions; self-reported awareness of antismoking advertisements; and sociodemographic characteristics. The survey data are weighted to reflect the state population of adults, adjusting for different probabilities of selection and survey nonresponse. The NY-ATS protocols were reviewed and approved by the sanctioned Institutional Review Boards of RTI International and the New York State Department of Health. The current analysis draws on media reach and quitline and NRT uptake among current smokers from survey years 2005–2008 (N = 5,109); in years before 2005 and after 2008, survey questions did not capture appropriate information for this analysis. Data on expenditures, budgets, and pack sales from NY TCP were also used. 

### 2.2. Defining Outcomes

**Figure 1:**
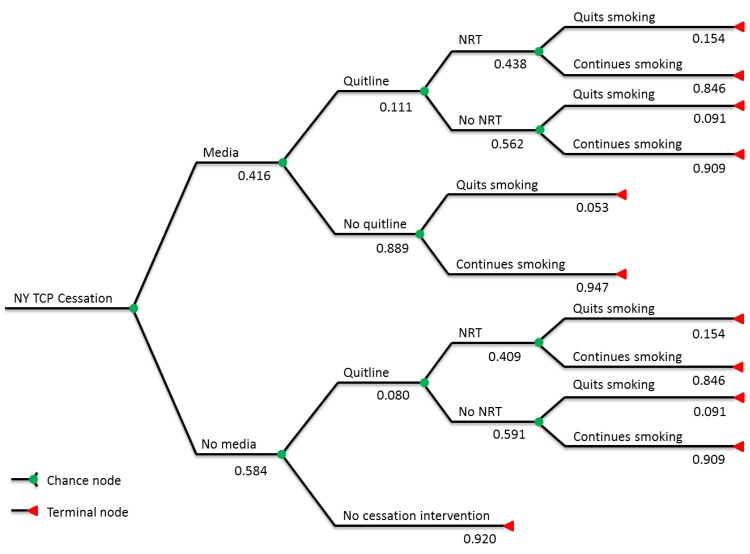
Simulation tree with branch expected values.

The first step in the analysis was to define outcomes for adult smokers in New York State. Analysis of 2005–2008 NY-ATS data provided rates for media awareness and quitline and NY TCP NRT utilization rates. These estimates, together with quit rates derived from the literature discussed in the Introduction, were used to sort adult smokers into six groups, based on the combination of interventions received (Figure 1); for those who received one or more kinds of intervention, additional quits induced were then estimated. The six groups are (1) media-aware smokers who call the quitline and receive NRT, (2) media-aware smokers who call the quitline but do not receive NRT, (3) media-aware smokers who do not call the quitline, (4) non-media-aware smokers who call the quitline and receive NRT, (5) non-media-aware smokers who call the quitline but do not receive NRT, and (6) smokers who are reached by none of the three interventions. Note that, because NY TCP distributes NRT only through the quitline, no groups received NRT without also receiving quitline counseling. Cessation rates were derived from weighted averages in the literature and assigned based on the interventions received. Because the literature has only examined changes in prevalence from media campaigns, or the degree to which media campaigns affect uptake rates in quit assistance, but not if media itself improves quit rates for those using further quit assistance, the assumption was made that quitline counseling and NRT would dominate; *i.e.*, media exposure is assumed to increase the probability of a successful quit in absence of further publicly-funded assistance, but that if quitline counseling or free NRT are used, successful quit probabilities are independent of media exposure.

### 2.3. Costs

Costs were computed from NY TCP expenditures and budgets. Although the simulation was designed based on 4 years (2005–2008) of NY TCP intervention reach and uptake estimates, the simulation is designed to estimate quits induced in a single year. While this would suggest the cost figure should correspondingly reflect a single year’s expenditures, we feel it is more accurate that cost figures reflect these outcomes taking place within the larger context of an ongoing program [4,7]. The NY TCP budget for financial year 2008 is, currently, the largest budget the program has received; thus, in the interest of keeping the net benefit estimate conservative, this budget was used as the basis of the overall cost figure. Thus, the simulation’s budget includes all expenditures for 2008, as well as portions of NY TCP expenditures from years before and after. Specifically, an arbitrary distribution was chosen incorporating 50% of expenditures from 2007 and 2009 and 25% of expenditures from 2006 and 2010. These proportions are necessarily by assumption, because neither data nor studies indicate how, in an ongoing program, expenditures from one year affect outcomes in any other year, but literature findings of an ongoing effect suggest it should not be assumed away. Thus, proportions were chosen that seemed reasonable for the simulation. This results in an overall cost figure of $204.1 million; of this, $46.7 million is for media, $9.6 million is for the quitline, and $20.9 million is for NRT, for a total of $77.2 million in cessation interventions. The remaining $126.9 million represents all other NY TCP expenditures, such as enforcement, administration, and other NY TCP programs (e.g., youth-targeted initiatives, community program support).

### 2.4. Benefits

The benefit of NY TCP intervention that was quantified is the smoking costs avoided due to NY TCP-induced quits. The basis of this figure is a CDC estimate of smoking-attributable mortality, years of potential life lost, and productivity losses; [17] in real 2011 U.S. dollars, this estimate comes to $12.47 per pack. In 2008, more than 540 million packs of cigarettes were sold in New York State, or about 11.4 cigarettes per day per smoker, consistent with other estimates in the literature. Studies indicate that quitters tend to smoke fewer cigarettes, prior to quitting, than those who do not quit [18,19]. Because these studies do not provide a specific relative level of consumption, incorporating this required us to assume one instead. In the absence of these estimates, the assumption was made that former smokers consumed at 80% of the level of continuing smokers, prior to quit; given the estimated adult smoker population and New York state cigarette sales in 2008, this comes to approximately 211 packs per year for continuing smokers and 169 packs per year for quitting smokers. Multiplying the consumption level for quitting smokers by damages per pack results in a gross benefit of $2,108 per quit per year of life without smoking, before any discounting or other modifications.

Studies indicate that, over time, health outcomes for smokers who quit can approach those of never-smokers [20]. However, these improved outcomes do not materialize to their full extent immediately [21]. Furthermore, benefits accrued from quitting are negatively associated with age (*i.e.*, the older a smoker is at time of permanent quit, the lower the improvement in life expectancy and health outcomes) [22,23]. Therefore, cumulative benefits of smoking were computed over 20 years; in addition to discounting, the reduced benefit, compared to never-smokers, and the time it takes for benefits to be realized were incorporated; a quadratic form was assumed, with benefits at 40% of the above figure in the first year and asymptotically approaching 67%. This yields a per-quit gross benefit of $20,011.53 over 20 years for a former smoker, from the time of quit.

Smoking cessation is characterized by frequent relapses; most successful quits follow multiple quit attempts that last anywhere from a few days to many months prior to relapse to smoking. Relapse rates are highest in the earliest stages of the quit attempt, and any benefits accumulated during these nonsmoking periods are effectively lost when smoking is resumed [20,24]. Hawkins *et al.* [20] found a 10-year relapse rate of 37.1% and negligible relapse rates after 10 years (1% or less). This relapse rate was used and did not include benefits from smokers that relapse.

Net benefit, in the model, was calculated by multiplying the number of additional quits induced (net of relapse) by the gross benefit per quit, yielding total gross benefit. The cost figure was then subtracted from gross benefit to find total net benefit. 

### 2.5. Model

The analysis was conducted using a tree-based Monte Carlo simulation, designed and run in the TreeAge software package. Figure 1 shows the tree, including expected values for each branch. All probabilities shown in the tree, as well as the 10-year relapse rate and background quit rate, are allowed to vary, using triangular distributions to account for uncertainty about the nature of the actual distributions. (See technical appendix for further details.) Quit rates shown for each intervention reflect the *additional* quits induced by said interventions (*i.e.*, they are the respective quit rate for each group less the background quit rate). For example, the expected value of the cessation rate for NRT recipients is 17.42%, but it is assumed that 2% of these recipients would have quit without intervention [25,26]; the benefits derive from the 15.42% of NRT recipients who quit, but who would not have without intervention. These quit rates do not reflect relapses, however; relapse is accounted for in actual benefit computation.

The analysis assumes a 3% discount rate, a conservative rate common in benefit-cost analysis, and a time horizon of 20 years. NY ATS data, combined with Census population estimates, yield an estimated 2008 New York adult smoker population of 2,582,969. A 2% background quit rate [25,26] (*i.e.*, quit rate absent any cessation intervention) was assumed, although, like all cessation rates in the simulation, some uncertainty is allowed. Note that the relationship between the background quit rate and net benefit is negative, all else held constant, whereas cessation assistance quit rates are positively associated with net benefit. This is because net benefit is dependent on how much cessation assistance increases quits over what would have happened without any cessation intervention. For example, holding the overall NRT quit rate at 17.42%, but increasing the background quit rate by one percentage point, to 3%, would result in the tree quit rate for NRT being 14.42% instead of 15.42%, resulting in fewer estimated additional quits due to NRT.

## 3. Results

**Figure 2:**
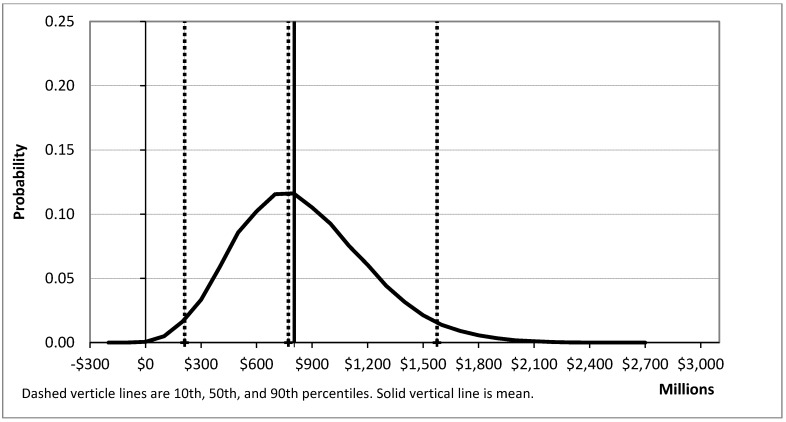
Net benefit estimation distribution.

**Figure 3:**
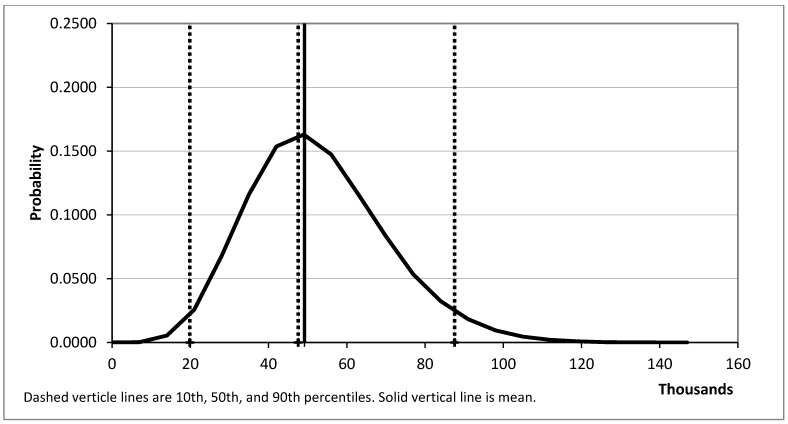
Additional quit estimate distribution.

Simulations were run to estimate additional quits (net of relapses to smoking) and net benefit. A total of 100,000 samples were run for each of these; in each sample, all distributions were allowed to vary. The results are summarized in Table 1 and Figure 2, Figure 3.

**Table 1:** Principle simulation results, statistical summary.

Statistic	Additional quits	Net benefit
**Mean**	**49,195**	**$803,770,725.66**
Std Dev	17,391	$349,691,254.29
Minimum	2,621	−$138,879,007.88
**2.5%**	**19,878**	**$211,089,884.14**
**Median**	**47,568**	**$771,864,156.51**
**97.5%**	**87,561**	**$1,575,068,586.97**
Maximum	143,258	$2,697,143,113.96

Running 100,000 trials (*i.e.*, simulation runs) for net benefit produces a mean of $804 million and a median of $772 million, with a 2.5 percentile value of $211 million and a 97.5 percentile value of $1,575 million. An additional 100,000 trials for additional quits produces a mean of 49,195 and a median of 47,568, with 2.5 percentile value of 22,749 and a 97.5 percentile value of 89,570. As shown in Figure 2, Figure 3, and reflected in the numbers above, the simulation parameters resulted in a small positive skew.

Our simulation results indicate that, given previous findings on the effectiveness of publicly-funded tobacco cessation assistance, NY TCP’s media campaigns, quitline counseling, and NRT most likely resulted in a substantial increase in the rate of successful quits, and provided net benefits, in the form of reduced health costs and improved productivity, in excess ofexpenditures. The 95% confidence interval of net benefits indicates that the benefit-cost ratio was likely at least 2:1, and more likely closer to 5:1, with cost figures that include not only the initiatives that drive the benefits but a number of other costs, many of which fund programs that may well generate additional benefits, such as youth-oriented programs to reduce initiation.

## 4. Discussion

The considerable variation in the results reflects the uncertainty of both intervention reach and cessation effects. Among interventions, media awareness is the most reliable figure; NY ATS data include multiple questions to discern whether respondents have seen the advertisement and recall content. Questions about quitline use, however, are much simpler and do not establish whether the respondent called only once, called several times, or made extensive use of the service. Similarly, questions about NRT do not extend to establishing compliance or length of use or how intensely quitline counseling was used in conjunction. 

Furthermore, because of survey limitations, cessation outcomes cannot be estimated from those data; thus, as mentioned above, quit rate estimates were derived from the literature instead. Because actual intervention-influenced cessation rates and even the background quit rate may vary considerably, appropriate degrees of uncertainty in the simulation are allowed for. Similarly, uncertainty around long-term relapse rates are allowed for; although estimates of short-term (*i.e.*, 1 year or less) relapse rates are reasonably well-established in the literature, studies on relapse over a longer time horizon are still relatively limited.

Despite the uncertainty reflected in the estimates, the incidence of negative net benefit in the simulation was extremely low (67 out of 100,000 trials, or 0.067%). This result suggests that NY TCP cessation initiatives generate enough benefit from those induced cessation attempts to offset not only their own costs, but also expenditures for the program as a whole. Given that cessation initiatives comprise only a fraction of overall NY TCP expenditures (indeed, even the media expenditures included above are not confined to cessation messages alone), and that a number of other initiatives may generate substantial benefit as well (e.g., reduced initiation, reduced consumption among continuing smokers), the results suggest a positive net benefit from NY TCP cessation interventions.

There are, however, notable limitations on the conclusions. Because actual cessation rates cannot be clearly established, a causal relationship cannot be established between intervention reach and cessation rates or control for non-NY TCP cessation aids or intervention (e.g., private media campaigns and advocacy, private counseling, over-the-counter NRT). Links cannot be established between particular levels of expenditure and intervention reach. Furthermore, disaggregating costs and benefits by intervention type is problematic; studies of introducing state-funded NRT to an established program with media and quitline components show that NRT itself will increase quitline use. Thus, while NY ATS data show a positive correlation between media awareness and both quitline and NRT use, these three interventions exist in a larger context of an ongoing program, and accurately predicting the effects of one in the absence of one or both of the others is not feasible with the data available.

That said, the results can be evaluated in another light. In 2008, more than 540 million packs of cigarettes were sold in New York State. That number dropped to 469 million in 2009, and to just under 408 million in 2010. In that short span of time, total cigarette consumption fell by over 14% per year, much higher than the simulation’s estimated adult cessation rate of about 5%, consistent with similar prior studies [27,28]. However, this reduction in observed consumption may be due to smokers reducing per-period cigarette consumption, lower initiation, higher cessation, cigarette purchases from across state lines or from tribal lands, or some combination of the four, and none of these possible causes can be isolated from the others, let alone estimate what portion represents NY TCP-induced cessation. Unemployment and reduced household income, due to the 2007–2009 recession and its ongoing effects, may also play a role, but studies indicate that unemployment does not appear to reduce smoking prevalence or tobacco consumption [29,30,31]. Nonetheless, the results do appear to provide some evidence of state-funded cessation intervention generating a positive net benefit, and future work can further establish the role of these programs in reducing smoking prevalence.

While there is little doubt that the reduction of tobacco consumption has significant public health benefits, estimation of the benefits from one specific cessation-oriented program is comparatively rare; cost-effectiveness is more commonly evaluated in the literature. Further, the approach used can serve as the basis for further work in evaluating and analyzing the effects of public health initiatives. There are multiple possibilities for further refinements, such as more precise interaction effects, additional data on uptake and reach (including distinguishing the cumulative effects possibly due to the presence of an ongoing program from the temporary effects of e.g., a specific short-term media campaign), and the implementation of additional outcomes (e.g., relapse and re-cessation, initiation, altered consumption levels).

## Supplementary Files

Supplementary File 1 Technical Supplementary (PDF, 48 KB)

## References

[1] Surgeon General’s Advisory Committee on Smoking and Health (1964). Report of the Advisory Committee to the Surgeon General of the Public Health Service.

[2] Centers for Disease Control and Prevention (2011). Vital signs: Current cigarette smoking among adults aged ≥18 years—United States, 2005–2010. MMWR.

[3] Centers for Disease Control and Prevention (1999). Best Practices for Comprehensive Tobacco Control Programs.

[4] Centers for Disease Control and Prevention (2007). Best Practices for Comprehensive Tobacco Control Programs.

[5] Woods S.S., Haskins A.E.  (2007). Increasing reach of quitline services in a US state with comprehensive tobacco treatment. Tob. Control.

[6] Kaufman A., Augustson E., Davis K., Finney Rutten L.J.  (2010). Awareness and use of tobacco quitlines: Evidence from the health information national trends survey. J. Health Commun..

[7] Chattopadhyay S., Pieper D.R. (2012). Does spending more on tobacco control programs make economic sense? An incremental benefit-cost analysis using panel data. Contemp. Econ. Policy.

[8] Farrelly M.C., Davis K.C., Nonnemaker J.M., Kamyab K., Jackson C. (2011). Promoting calls to a quitline: Quantifying the influence of message theme, strong negative emotions and graphic images in television advertisements. Tob. Control.

[9] Farrelly M.C., Hussin A., Bauer U.E.  (2007). Effectiveness and cost effectiveness of television, radio and print advertisements in promoting the New York smokers’ quitline. Tob. Control.

[10] Mosbaek C.H., Austin D.F., Stark M.J., Lambert L.C.  (2007). The association between advertising and calls to a tobacco quitline. Tob. Control.

[11] An L.C., Schillo B.A., Kavanaugh A.M., Lachter R.B., Luxenberg M.G., Wendling A.H., Joseph A.M. (2006). Increased reach and effectiveness of a statewide tobacco quitline after the addition of access to free nicotine replacement therapy. Tob. Control.

[12] Fellows J.L., Bush T., McAfee T., Dickerson J.  (2007). Cost effectiveness of the Oregon quitline “free patch initiative”. Tob. Control.

[13] Hollis J.F., McAfee T.A., Fellows J.L., Zbikowski S.M., Stark M., Riedlinger K.  (2007). The effectiveness and cost effectiveness of telephone counselling and the nicotine patch in a state tobacco quitline. Tob. Control.

[14] Tinkelman D., Wilson S.M., Willett J., Sweeney C.T. (2007). Offering free NRT through a tobacco quitline: Impact on utilisation and quit rates. Tob. Control.

[15] Stead L.F., Perera R., Lancaster T.  (2007). A systematic review of interventions for smokers who contact quitlines. Tob. Control.

[16] Cummings K.M., Fix B.V., Celestino P., Hyland A., Mahoney M., Ossip D.J., Bauer U.  (2010). Does the number of free nicotine patches given to smokers calling a quitline influence quit rates: Results from a quasi-experimental study. BMC Public Health.

[17] Centers for Disease Control and Prevention (2006). Sustaining State Programs for Tobacco Control.

[18] Etter J.F. (2004). Associations between smoking prevalence, stages of change, cigarette consumption, and quit attempts across the United States. Prev. Med..

[19] White V.M., Gilpin E.A., White M.M., Pierce J.P. (2005). How do smokers control their cigarette expenditures?. Nicotine Tob. Res..

[20] Hawkins J., Hollingworth W., Campbell R. (2010). Long-term smoking relapse: A study using the British household panel survey. Nicotine Tob. Res..

[21] U.S. Department of Health and Human Services (1990). The Health Benefits of Smoking Cessation: A Report of the Surgeon General.

[22] Doll R., Peto R., Boreham J., Sutherland I. (2004). Mortality in relation to smoking: 50 years’ observations on male British doctors. BMJ.

[23] Streppel M.T., Boshuizen H.C., Ocke M.C., Kok F.J., Kromhout D. (2007). Mortality and life expectancy in relation to long-term cigarette, cigar and pipe smoking: The zutphen study. Tob. Control.

[24] Krall E.A., Garvey A.J., Garcia R.I. (2002). Smoking relapse after 2 years of abstinence: Findings from the VA normative aging study. Nicotine Tob. Res..

[25] Cornuz J., Pinget C., Gilbert A., Paccaud F. (2003). Cost-effectiveness analysis of the first-line therapies for nicotine dependence. Eur. J. Clin. Pharmacol..

[26] Javitz H.S., Swan G.E., Zbikowski S.M., Curry S.J., McAfee T.A., Decker D.L., Patterson R., Jack L.M. (2004). Cost-effectiveness of different combinations of bupropion SR dose and behavioral treatment for smoking cessation: A societal perspective. Am. J. Manag. Care.

[27] Levy D.T., Friend K. (2002). A simulation model of policies directed at treating tobacco use and dependence. Med. Decis. Making.

[28] Levy D.T., Mabry P.L., Graham A.L., Orleans C.T., Abrams D.B. (2010). Exploring scenarios to dramatically reduce smoking prevalence: A simulation model of the three-part cessation process. Am. J. Public Health.

[29] Goel R.K. (2008). Unemployment, insurance and smoking. Appl. Econ..

[30] Lee A.J., Crombie I.K., Smith W.C., Tunstall-Pedoe H.D. (1991). Cigarette smoking and employment status. Soc. Sci. Med..

[31] Novo M., Hammarström A., Janlert U. (2000). Smoking habits—A question of trend or unemployment? A comparison of young men and women between boom and recession. Public Health.

