# Supplementary material for: New York Tobacco Control Program Cessation Assistance: Costs, Benefits, and Effectiveness
Source: Int J Environ Res Public Health. 2013 Mar 13;10(3):1037–47. doi: 10.3390/ijerph10031037 (PMC3709302; doi:10.3390/ijerph10031037)
Supplement: Supplementary File 1 — Technical Supplementary (PDF, 48 KB) [file ijerph-10-01037-s001.pdf]

Technical Supplementary

A.1. Relative Consumption

In our paper, we derive a benefit figure for smoking cessation of $20,017.40 over 20 years, including discounting. Part of the basis for this benefit figure is in our assumption that smokers who quit consume at a lower level (*i.e.*, smoke fewer cigarettes per year) than those who do not. We hold total consumption constant while varying this, so that while average consumption remains at 209.17 packs per year, continuing smokers consume more than that, and quitting smokers consume less. To derive our figures of 211.36 packs per year for continuing smokers and 169.09 packs per year for quitting smokers, we start by using the expected values for all intervention reach and uptake rates and quit rates to arrive at an expected value for total quits of 133,818. These are adult smokers who have made a quit attempt and successfully ceased smoking for 12 months and includes both background quits and quits aided by one or more NY TCP cessation intervention. First, we define some variables and constants (where constants are denoted by bars):

$$C_C = Packs\ per\ year\ for\ all\ continuing\ smokers$$

$$C_Q = Packs\ per\ year\ for\ all\ quitting\ smokers$$

$$\overline{P}_C = Population\ of\ continuing\ smokers$$

$$\overline{P}_Q = Population\ of\ quitting\ smokers$$

We can then define another constant and two more variables:

$$\overline{\beta} = \frac{\overline{P}_C}{\overline{P}_Q}$$

$$\gamma = \frac{C_C}{C_Q}$$

$$\alpha \frac{C_C}{\overline{P}_C} = \frac{\gamma C_Q}{\overline{\beta}\overline{P}_Q}$$

The variable α is relative consumption; for example, if $\alpha = 1$, then $\gamma = \beta$ and consumption levels are equal between the two groups. As stated above, we are holding the total population as well as total consumption constant.

$$\overline{C} = C_C + C_Q$$

$$\overline{P} = \overline{P_C} + \overline{P_Q}$$

Therefore, if we allow α to vary, γ must also vary. If we want to set α to 0.5, so that quitting smokers consumed at half the intensity of continuing smokers prior to quitting, then we must find a value of γ that holds constant overall consumption. Rearranging the above equations for β and γ yields the following:

$$S_Q = \frac{S_C}{\beta}$$

$$C_Q = \frac{C_C}{\gamma}$$

This, in turn, implies the following:

$$\alpha \frac{C_C}{P_C} = \frac{\frac{C_C}{\gamma}}{\frac{P_C}{\beta}}$$

Cancelling and rearranging terms yields the following:

$$\gamma = \frac{\beta}{\alpha}$$

The constant β, using expected values, is approximately 18.86. Thus, if, as in our example, we set the consumption level for quitting smokers at half that of continuing smokers, then $\gamma \approx 37.72$. However, in our evaluation framework, this variable is not easily interpretable; instead, we find a formula to directly calculate consumption by both groups. We begin by noting:

$$C_Q = \frac{C_C}{\gamma}$$

$$\Rightarrow \overline{C} = C_C + \frac{C_C}{\gamma}$$

Using some substitution and rearranging terms, we find the following:

$$C_C = \frac{\overline{C}}{\left(1 + \frac{\alpha}{\beta}\right)}$$

Put more simply, we can therefore compute packs per year consumed by continuing smokers from the ratio of continuing to quitting smokers, β, the assumed relative consumption level of quitting smokers, α, and total consumption. We can then compute packs per year per continuing smoker and packs per year per quitting smoker, and verify that relative consumption is at our desired level and that total consumption is constant. It is worth noting that changes in relative consumption, as might be surmised from the above, lead to far more dramatic changes in per-smoker consumption among quitting smokers than among continuing smokers.

A.2. Cost and Benefit Computation

As described in the main body of the text, we use Centers for Disease Control and Prevention (CDC) smoking damage estimates as the basis for our estimate of cessation benefits. These are expressed on a per-pack basis; using packs per year for quitting smokers computed above, we can in turn compute the initial figure for damages per year per smoker described above. We then compute cumulative benefit over the twenty-year time horizon, incorporating discounting and our assumptions about the shape of the benefit curve (detailed in the main paper).

$$\sum_{i=1}^{20} \frac{\$2{,}108.20 \times [\sum_{k=1}^{i} 0.4^k]}{(1.03)^i} = \$20{,}017.40$$

Gross benefit is computed in the simulation by estimating quits at one year, and then multiplied by $(1 - relapse\ rate)$ to arrive at post-relapse gross benefit. As mentioned above, relapse rate, like quit and intervention reach rates, is allowed to vary according to our uncertainty assumptions.

A.3. Uncertainty

As described above, we allow for considerable uncertainty in our results, reflecting the uncertainty of our assumptions about intervention reach and uptake rates, quit rates, and relapse rates. We use triangular distributions in the simulation, since, while we have variously robust measurements of all of these rates, there is less certainty about the shape of the distributions. In our final model, all distributions were symmetric, centered on the expected value as the mode.

For relapse rates, the background quit rate, and intervention reach and uptake rates, the minima and maxima are computed as follows:

$$a = 0.5 \cdot rate$$

$$b = 1.5 \cdot rate$$

For intervention-driven quit rates, however, we chose to allow these rates to be as low as the background quit rate. Therefore, to maintain symmetry, the extrema are computed as follows:

$$a = Background\ quit\ rate$$

$$b = (2 \cdot Intervention\ quit\ rate) - Background\ quit\ rate$$

Table A-1 presents the full list of distribution parameters, along with simulation standard deviations.

Table A-1. Uncertainty parameters and simulation statistics.

Distribution	Minimum (a)	Mode (c) (*i.e.*, E(x))	Maximum (b)	SD, net benefit simulation	SD, additional quits simulation
Media awareness	0.1964	0.3928	0.5893	0.0802	0.0800
Quitline use, media-aware smokers	0.0868	0.1736	0.2604	0.0354	0.0355
NRT use, media-aware smokers	0.2149	0.4299	0.6448	0.0879	0.0876
Quitline use, non-media-aware smokers	0.0489	0.0979	0.1468	0.0200	0.0200
NRT use, non-media-aware smokers	0.1709	0.3419	0.5128	0.0699	0.0699
Quit rate, media-only smokers	0.0200	0.0725	0.1250	0.0215	0.0215
Quit rate, quitline-using smokers, no NRT	0.0200	0.1111	0.2022	0.0371	0.0371
Quit rate, smokers using NRT	0.0200	0.1742	0.3283	0.0629	0.0631
Background quit rate	0.0100	0.0200	0.0300	0.0041	0.0041
Relapse rate	0.1855	0.3710	0.5565	0.0756	0.0757

Note: NRT = nicotine replacement therapy; SD = standard deviation.

As a robustness and sensitivity test, we also implemented versions of the model that incorporated right-skewed quit rate distributions (*i.e.*, a lower mode and higher maximum), such that the expected value was the same as in our final model. The output of this model was not effectively different; the overall range was larger, but measures of central tendency and confidence intervals yielded similar results to our final model.
